# Recruitment of *Arabidopsis* RNA Helicase AtRH9 to the Viral Replication Complex by Viral Replicase to Promote Turnip Mosaic Virus Replication

**DOI:** 10.1038/srep30297

**Published:** 2016-07-26

**Authors:** Yinzi Li, Ruyi Xiong, Mark Bernards, Aiming Wang

**Affiliations:** 1 London Research and Development Centre, Agriculture and Agri-Food Canada, London, Ontario, N5V 4T3, Canada; 2 Department of Biology, Western University, London, Ontario, N6A 5B7, Canada

## Abstract

Positive-sense RNA viruses have a small genome with very limited coding capacity and are highly dependent on host components to fulfill their life cycle. Recent studies have suggested that DEAD-box RNA helicases play vital roles in many aspects of RNA metabolism. To explore the possible role of the RNA helicases in viral infection, we used the *Turnip mosaic virus* (TuMV)-*Arabidopsis* pathosystem. The *Arabidopsis* genome encodes more than 100 putative RNA helicases (AtRH). Over 41 *Arabidopsis* T-DNA insertion mutants carrying genetic lesions in the corresponding 26 *AtRH* genes were screened for their requirement in TuMV infection. TuMV infection assays revealed that virus accumulation significantly decreased in the *Arabidopsis* mutants of three genes, *AtRH9*, *AtRH26*, and *PRH75*. In the present work, *AtRH9* was further characterized. Yeast two-hybrid and bimolecular fluorescence complementation (BiFC) assays showed that AtRH9 interacted with the TuMV NIb protein, the viral RNA-dependent RNA polymerase. Moreover, the subcellular distribution of AtRH9 was altered in the virus-infected cells, and AtRH9 was recruited to the viral replication complex. These results suggest that *Arabidopsis* AtRH9 is an important component of the TuMV replication complex, possibly recruited via its interaction with NIb.

RNA viruses have small and compact genomes with limited coding capacity and live exclusively in their host cells. During the long-time co-evolution with the hosts, they have acquired the ability to recruit host proteins and reprogram host metabolites to fulfill their life cycle^1^,^2^,^3^,^4^,^5^,^6^. In the last decade, extensive efforts have been made to identify and characterize a number of important host factors recruited for virus infection, since their disruption may provide novel protection strategies for crop plants. These host factors play versatile roles during plant RNA virus replication^2^,^5^,^7^, including (1) assistance in assembly of the viral replication complex (VRC) and cellular membrane remodelling; (2) recruitment of viral proteins and template RNA to the VRC; (3) regulation of the switch from viral genome translation to replication; (4) participation in the intracellular transport of viral proteins and viral RNA; and (5) facilitating folding of viral proteins as protein chaperones.

Potyviruses, belonging to the *Potyviridae*, represent the largest group of known plant viruses and include many agriculturally and economically important pathogens such as *Turnip mosaic virus* (TuMV)^8^,^9^. As one of the most prevalent viral pathogens, TuMV infects a wide range of plant species, mostly (although not exclusively) in the *Brassicaceae*^10^,^11^. Like other potyviruses, TuMV has a positive-sense, single-stranded RNA genome, which is covalently linked with a viral genome-linked protein VPg at the 5′ end, and contains a 3′-polyadenylated [poly(A)] tail. The viral genomic RNA codes for a long open reading frame (ORF) and another relatively short ORF that results from RNA polymerase slippage in the P3 coding sequence^12^,^13^,^14^,^15^. The two polyproteins translated from these two ORFs are ultimately cleaved by three viral proteinase domains into 11 mature proteins: P1, helper component protease (HC-Pro), P3, P3N-PIPO, 6K1, cylindrical inclusion (CI) protein, 6K2, VPg, nuclear inclusion a (NIa), nuclear inclusion b (NIb), and capsid protein (CP)^16^.

The VRC of all positive-sense RNA viruses is intimately associated with intracellular membranous structures^17^. A variety of host proteins are recruited to form the VRC^1^,^2^,^4^,^5^. For example, the host proteins of the TuMV VRC include proteins such as eukaryotic translation initiation factors (eIFs)^18^,^19^, a cysteine-rich protein PVIP^20^, heat shock cognate 70-3 (Hsc70-3)^21^, poly(A) binding protein (PABP)^22^, eukaryotic elongation factor 1A (eEF1A)^23^, *Arabidopsis* RNA helicase 8 (AtRH8)^24^ and DNA-binding protein phosphatase 1 (DBP1)^25^.

DEAD-box RNA helicases (DDXs) represent a large family of RNA helicases (RHs) that have been implicated in almost every step of RNA metabolism from transcription, mRNA splicing and translation, RNA modification and transport, ribosome biogenesis and RNP complex assembly to mRNA degradation^26^. DDXs utilize ATP to catalyze the separation of RNA duplexes and promote the structural rearrangement of RNA-protein complexes^27^. The name of the family was derived from the highly conserved amino acid sequence D-E-A-D (Asp–Glu–Ala–Asp) in motif II^28^ of the protein. In a seminal work, Noueiry and colleagues showed that the yeast gene *DED1*, encoding a human DDX3-like RNA helicase, is indispensable for the translation of *Brome mosaic virus* (BMV)^29^. Consistently, downregulation of *DED1* negatively affects *Tomato bushy stunt virus* (TBSV) infection by inhibiting the accumulation of virus-encoded replication proteins^30^,^31^. The *DED1*-encoded protein Ded1p is also required for the replication of *Flock house virus* (FHV)^32^. The yeast DDX Ded1 and its plant ortholog AtRH20 suppress viral RNA recombination and maintain viral genome integrity^33^. Similar to Ded1p, another DDX Dbp2p (the homolog of the human p68 protein) promotes tombusvirus positive-strand synthesis in yeast^31^. Dbp2p binds to the 3′ end of the TBSV negative-sense RNA and unwinds the local secondary structure^31^. In plants, AtRH20, an RNA helicase in *Arabidopsis* sharing high sequence similarity with Dbp2, can stimulate TBSV positive-sense RNA synthesis, suggesting RNA helicases in plants may assist viral replication in a similar manner^31^. Recently, two additional cellular RNA helicases, e.g., the eIF4AIII-like yeast FAL1 and the DDX5-like Dbp3 and their orthologs in *Arabidopsis,* AtRH2 and AtRH5, have been shown to be present in the tombusvirus VRCs^34^. In the case of potyviruses, an *Arabidopsis* DEAD-box RNA helicase, AtRH8 and a *Prunus persica* DDX-like protein, PpDDXL, have been identified to interact with potyviral VPg. AtRH8 co-localizes with the viral replication vesicles, suggesting AtRH8 is likely involved in viral genome translation and replication^24^. These studies demonstrate the essential roles of DDXs in virus infections.

The *Arabidopsis* genome encodes more than 100 putative DDXs (AtRH)^35^. To further identify AtRHs required for potyviral infection and explore their possible roles therein, we used *Arabidopsis* mutant plants generated by T-DNA insertion. Over 41 *Arabidopsis* T-DNA insertion mutants corresponding to 26 *AtRH* genes were screened for their resistance to TuMV infection. We found that TuMV accumulation was inhibited in *Arabidopsis* mutants of three *RH* genes *AtRH9*, *AtRH26* and *PRH75*. Herein we describe our characterization of *Arabidopsis AtRH9* and present evidence that AtRH9 interacts with TuMV NIb and is recruited to the VRC to promote TuMV infection.

## Results

### Screening for *Arabidopsis AtRH* gene mutants resistant to TuMV infection

**Figure 1:**
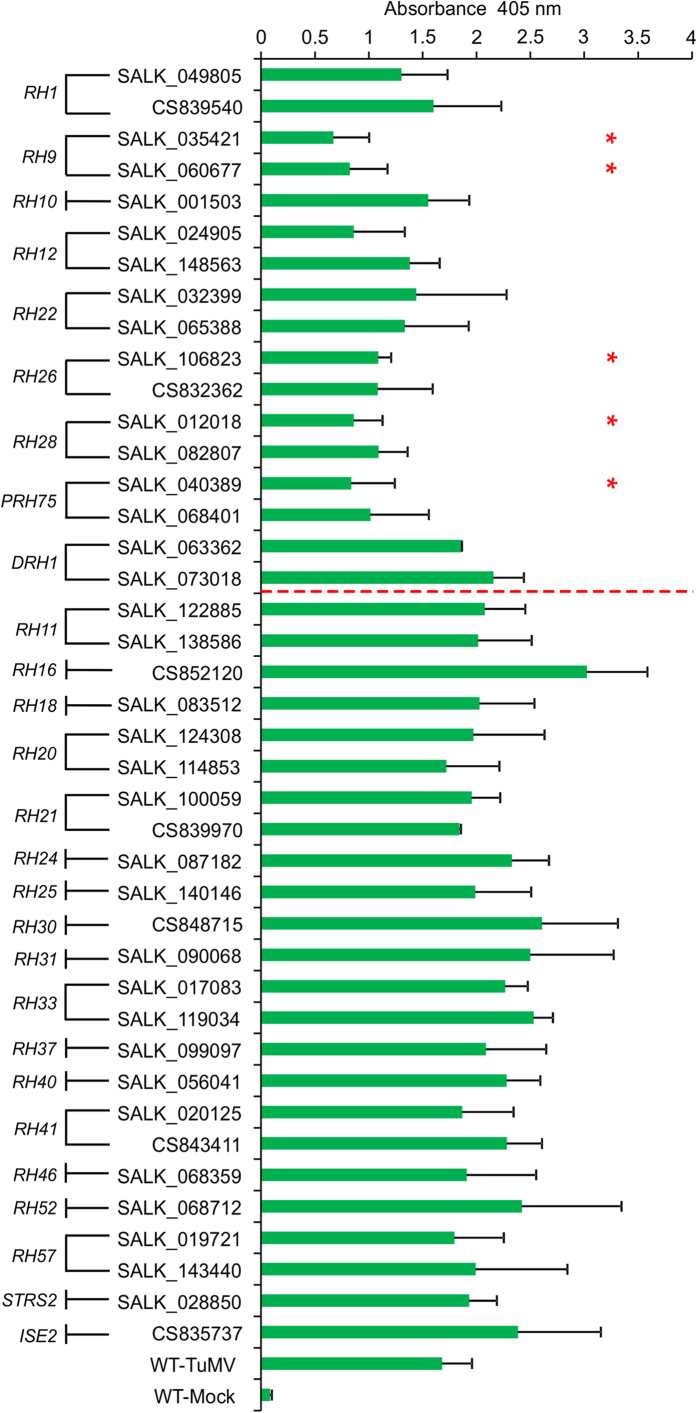
ELISA Analysis of *Arabidopsis atrh* T-DNA Mutants. A total of 41 homozygous *Arabidopsis* T-DNA insertion mutant lines corresponding to 26 *AtRH* genes were selected for TuMV infection assay. The *Arabidopsis atrh* T-DNA insertion mutants and WT plants were mechanically inoculated with TuMV. ELISA analysis was used to determine the accumulation of TuMV CP in *atrh* T-DNA mutants and WT plants 10 days post inoculation (dpi). Extracts from newly-emerged leaves of TuMV-infected individual plants were subjected to ELISA using TuMV CP-specific antibody. The x-axis represents ELISA values. Error bars represent standard deviation (n ≥ 5). Asterisk indicates significantly different from WT plants (unpaired two-tailed Student’s t test, p < 0.05).

Previous studies predicted that the *Arabidopsis* genome contained 53 *AtRH* genes^36^,^37^. A relatively more recent study suggested a total of 113 putative helicase genes encoded by the *Arabidopsis* genome^35^. The majority of them are ‘computer predicted putative helicases’, and only a few have been experimentally confirmed to have helicase activity. The biological functions of RHs in biological processes are poorly understood in general. To elucidate the role of *AtRH* genes in viral infection, *Arabidopsis* T-DNA insertion lines corresponding to 42 *AtRH* genes were selected from the TAIR database. These genes encode the proteins that are either related to eIF4A^24^ or have possible functions in the formation of plasmodesmata (PD)^38^ or stress response regulation^39^. Seed stocks of 128 *Arabidopsis* T-DNA insertion lines corresponding to these 42 *AtRH* genes were obtained from the Arabidopsis Biological Resource Center (ABRC). Mutant and insertion information was obtained from the Salk Institute Genomic Analysis Laboratory website (http://signal.salk.edu/). Genotyping identified a total of 53 homozygous *Arabidopsis* T-DNA insertion lines corresponding to 26 *AtRH* genes. Of these, we chose 41 homozygous lines with T-DNA insertions in either an exon or the 5′ UTR region for TuMV infection assays (Table 1). The 41 selected *Arabidopsis* T-DNA insertion lines and wild-type (WT) control plants were rub-inoculated with TuMV, followed by observation of disease symptoms. Newly-emerged leaves from systemically TuMV-infected T-DNA mutant plants and WT were sampled and assayed for viral CP accumulation at 10 days post inoculation (dpi) by Enzyme-linked Immunosorbent Assay (ELISA) (Fig. 1).

Among the 41 T-DNA lines, 18 *AtRH* gene mutants, corresponding to the genes *DRH1*, *AtRH11*, *AtRH16*, *AtRH18*, *AtRH20*, *AtRH21*, *AtRH24*, *AtRH30*, *AtRH31*, *AtRH33*, *AtRH37*, *AtRH40*, *AtRH41*, *AtRH46*, *AtRH53*, *AtRH57*, *STRS1*, and *STRS2*, displayed higher (albeit not statistically significant) CP accumulation than WT plants. In these mutants, TuMV infection induced severe phenotypes, including stunted growth, yellowish and curled leaves, chlorotic and mosaic lesions on leaves, similar to the symptoms in TuMV-infected WT (data not shown). In contrast, the TuMV CP accumulation level in T-DNA insertion lines of *AtRH9*, *AtRH26* and *PRH75* were significantly lower than that observed in WT plants. These three mutants, i.e., *atrh9*, *atrh26* and *prh75*, exhibited attenuated symptoms in the systemically-infected plants. Apparently, TuMV infection in these mutants was affected. Intriguingly, ELISA results also revealed that TuMV CP levels were reduced in newly-emerged leaves of T-DNA insertion lines of *AtRH1, AtRH10, AtRH12, AtRH22* and *AtRH28* relative to WT plants. But the reduction did not reach significant levels. Altogether this screen identified the biological relevance of three *AtRH* genes, i.e., *AtRH9, AtRH26* and *PRH75*, to TuMV infection.

### The accumulation of TuMV was reduced in *atrh9* mutant plants

Of the three gene mutants with resistance to TuMV infection, the two *atrh9* T-DNA insertion lines SALK_035421 and SALK_060677 showed the largest reduction in CP accumulation. Based on information from the *Arabidopsis* database, SALK_035421 contains a T-DNA insertion in the 6^th^ exon, whereas SALK_060677 has a T-DNA in the 3′ UTR region (Supplemental Fig. 1a,b,d). As SALK_035421 was confirmed to be a true knockout mutant^40^, we focused on this mutant. The *AtRH9* gene (also At3g22310) encodes an RH that was previously designated Putative Mitochondrial RNA Helicase1 (PMH1), and SALK_035421 was named *pmh1-1*^41^. In this study, to be consistent with the gene name *AtRH9* in the *AtRH* family^37^, we renamed the T-DNA insertion line SALK_035421 as *atrh9*. A SALK_035421 homozygous insertion line was obtained from ABRC and the homozygous T-DNA insertion was verified using PCR analysis (Supplemental Fig. 1b). Loss of transcripts in *atrh9* was confirmed by reverse transcription (RT)-PCR analysis using *AtRH9*-specific primers (Supplemental Fig. 1c), consistent with the published data^40^.

**Figure 2:**
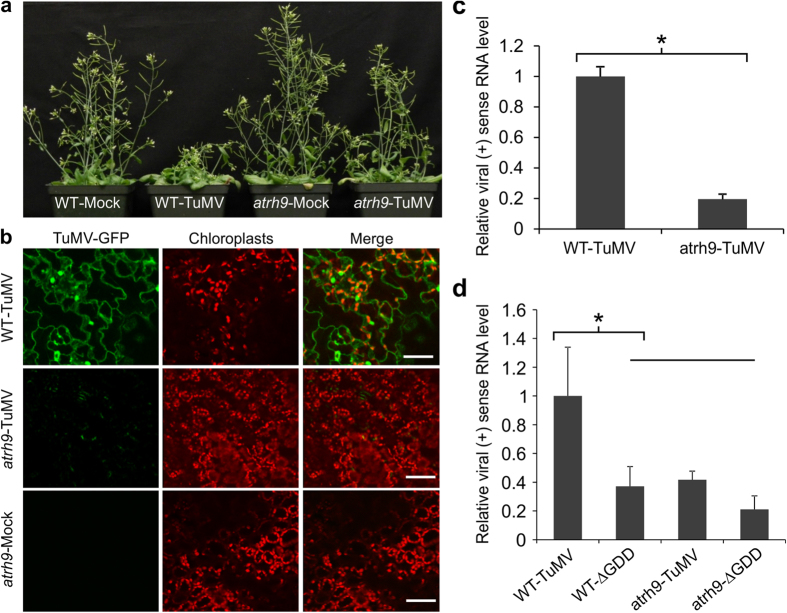
TuMV Accumulation in *atrh9* Mutant Plants. (**a**) Three-week-old *Arabidopsis atrh9* mutants (SALK_035421) and WT plants were agroinfiltrated with TuMV-GFP infectious clone. Phenotypes of TuMV-infected *atrh9* mutants and WT plants. Images were taken 10 days post agroinfiltration (dpa). Mock, inoculated with buffer; TuMV, inoculated with TuMV. (**b**) Newly-emerged leaves were observed by confocal microscopy 10 dpa, and representative images are shown. Mock, *atrh9* mutants and WT plants were agroinfiltrated with buffer. TuMV-GFP, green fluorescence emissions; Chl, chloroplast autofluorescence. Bars, 50 μm. (**c**) Relative fold changes of TuMV viral genomic RNA in *atrh9* mutant plants were determined by real-time RT-PCR at 10 dpa. RNA was extracted from newly-emerged leaves of infected individual plants. Three independent experiments, each consisting of three biological replicates were carried out for quantification analysis. Each value was normalized against *Actin2* transcripts in the same sample. The values are presented as means of fold changes relative to WT. Error bars represent standard deviation (n = 9). Statistically significant difference from WT plants, determined by an unpaired two-tailed Student’s t test, is indicated. *p < 0.05. (**d**) Detection of TuMV viral RNA by qRT-PCR in WT and *atrh9* mutant plants agroinfiltrated with a TuMV infectious clone (TuMV) and a replication-defective mutant (∆GDD). Both clones contain a separate expression cassette allowing transcript of mCherry transcripts in plant cells. RNA was purified from agroinfiltrated local leaves 54 hours post agroinfiltration (hpa). mCherry transcript was used as an internal control. The values are presented as means of fold change relative to WT. Error bars denote standard deviation of three biological replicates. Statistically significant differences from WT plants, determined by an unpaired two-tailed Student’s t test, are indicated. *p < 0.05.

Under standard growth conditions, *atrh9* mutant plants displayed no abnormal phenotypes distinguishable from *Arabidopsis* WT plants (Fig. 2a). In contrast to the severe TuMV symptoms (necrosis, chlorotic leaves and dwarfing) observed in WT plants, *atrh9* mutant plants displayed minor symptoms, such as curled bolts and slightly shorter in height (Fig. 2a). To confirm *atrh9* was resistant to TuMV, three-week-old *atrh9* mutants and WT plants were agroinfiltrated with a GFP-tagged TuMV infectious clone (TuMV-GFP). TuMV infection was monitored by confocal microscopy. In all three independent experiments, strong signals of GFP fluorescence were observed in the newly-emerged leaves of infected WT plants, whereas only weak and scattered GFP fluorescence was detected in *atrh9* mutant plants 10 days post agroinfiltration (dpa) (Fig. 2b). Quantitative RT-PCR (qRT-PCR) was carried out to quantify TuMV accumulation. Consistent with the observed reduction in GFP fluorescence, we observed a sharp decline of viral genomic RNA accumulation in *atrh9* mutant plants. As shown in Fig. 2c, TuMV viral accumulation in the mutant underwent a substantial decrease by 80% with respect to that in WT plants at 15 dpa.

To determine if viral replication is affected in the primarily infected cells in *atrh9* plants, a TuMV infectious clone containing a separate expression cassette for expression of mCherry and its corresponding replication-defective mutant (∆GDD) were agroinfiltrated into leaves of *Arabidopsis* WT and *atrh9* mutant plants. As potyviral intercellular movement would not occur until 96 hours post agroinfiltration (hpa)^42^, total RNA was isolated from the agroinfiltrated leaves at 54 hpa, and subjected to qRT-PCR analysis to determine viral RNA accumulation using mCherry as an internal control. It was found that viral genomic RNA in *atrh9* mutant plants was significantly lower than that in WT plants (Fig. 2d). Although it was slightly higher than that in both *atrh9* and WT *Arabidopsis* plants agroinfiltrated with the replication-defective TuMV ∆GDD clone, there was no significant difference (Fig. 2d). Taken together, these results suggest that AtRH9 is a host factor promoting TuMV replication.

### Silencing *AtRH9* in *Arabidopsis* results in partial resistance to TuMV infection

To further confirm the involvement of *AtRH9* in TuMV infection, a *Tobacco rattle virus* (TRV)-based virus-induced gene silencing (VIGS) system was employed to silence *AtRH9* expression in *Arabidopsis*. A cDNA fragment of *AtRH9* was cloned into a pTRV2-derived vector to produce pTRV2-AtRH9. *Arabidopsis* WT seedlings were co-agroinfiltrated with the vectors pTRV2-AtRH9 and pTRV1. A bleaching phenotype was observed in control plants 12 days after co-agroinfiltration with pTRV2-PDS and pTRV1, indicating VIGS was established. At this time point, the TuMV infection assay was applied on *AtRH9*-downregulated plants (treated with pTRV2-AtRH9 and pTRV1) as well as WT plants (treated with buffer) and negative control plants (treated with empty pTRV vectors). qRT-PCR was performed to evaluate *AtRH9* expression and TuMV accumulation at 15 dpi (Supplemental Fig. 2). The severely reduced level of the *AtRH9* transcript in *AtRH9*-downregulated plants was coupled with partial resistance to TuMV infection (Supplemental Fig. 2). Consistent with the results from TuMV infection assays on the *atrh9* mutant plants, these data reaffirm our hypothesis that downregulation of *AtRH9* effectively inhibits TuMV infection in *Arabidopsis*.

### AtRH9 is differentially distributed in TuMV-infected in *Nicotiana benthamiana* leaf cells, and co-localized with the VRCs

**Figure 3:**
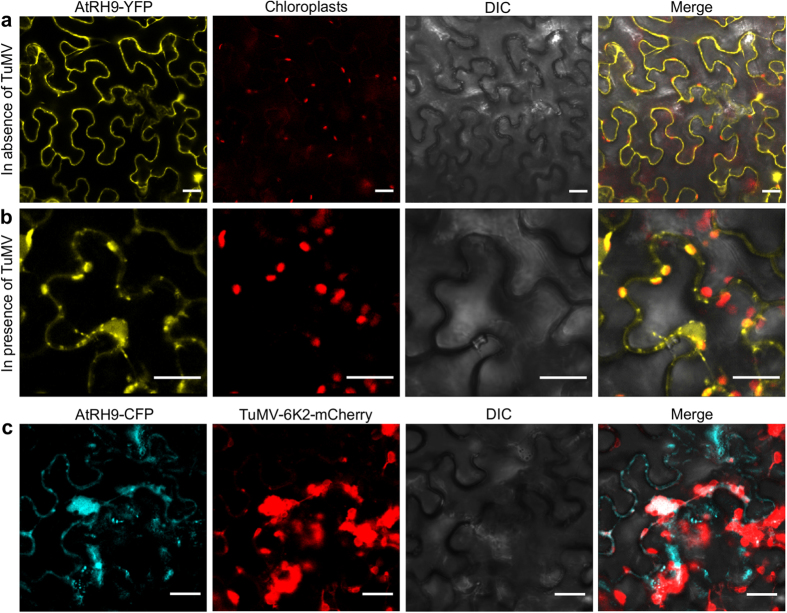
Altered Localization of AtRH9 in *Arabidopsis* during TuMV Infection. (**a**) Transient expression of AtRH9-YFP in *N. benthamiana* leaf epidermal cells. YFP fluorescence was observed using a confocal microscope 48 hours post agroinfiltration (hpa). (**b**) AtRH9-YFP was expressed in *N. benthamiana* leaves infected by TuMV::6K2-mCherry. Localization of AtRH9 is associated with chloroplasts in the course of viral infection at 72 hpa. Bars, 20 μm. (**c**) Transient expression of AtRH9-CFP in *N. benthamiana* leaves infected by TuMV::6K2-mCherry. CFP fluorescence was observed using a confocal microscope 72 hpa. AtRH9-CFP was observed mainly with irregularly-shaped globular-like aggregations from red fluorescence from TuMV VRCs (6K2-mCherry). DIC, differential interference contrast. Bars, 20 μm.

To gain insight into the molecular function of AtRH9 in TuMV infection, subcellular localization of AtRH9 was performed. A translational fusion of AtRH9 with YFP controlled by the CaMV 35S promoter was transiently expressed in *N. benthamiana* leaf epidermal cells via agroinfiltration. Subcellular localization of fusion proteins was monitored using a Leica TCS SP2 inverted confocal microscopy at 48 hpa. Consistent with a previous study^41^, most of the AtRH9-YFP was evenly distributed in the cytoplasm of *N. benthamiana* leaf cells without TuMV infection (Fig. 3a). In contrast, in TuMV-infected cells, AtRH9-YFP was found to form punctate and irregular-shaped aggregations, many of which were associated with chloroplasts (Fig. 3b). Thus, the distribution pattern of AtRH9 in TuMV-infected leaf cells was clearly different from that in the leaf cells free from TuMV infection.

Our previous studies have shown that 6K2-induced chloroplast-associated vesicles are the sites for potyviral replication^43^,^44^. These 6K2-labelled structures contain double-stranded replicative RNA intermediates, viral replicase proteins and many host factor proteins^43^,^45^,^46^,^47^,^48^. To determine if AtRH9 co-localizes with the chloroplast-associated 6K2 vesicles during TuMV infection, AtRH9-CFP was transiently expressed in *N. benthamiana* leaf cells infected by a TuMV infectious clone (TuMV::6K2-mCherry), which produces red-fluoresce labelled 6K2 vesicles during infection. AtRH9-CFP co-localized with 6K2-induced irregular-shaped aggregations in the cytoplasm of TuMV-infected leaf cells (Fig. 3c). These results indicated that AtRH9 was recruited to the VRC during TuMV replication, suggesting a functional role of AtRH9 in viral proliferation.

### AtRH9 interacts with TuMV RNA-dependent RNA polymerase

**Figure 4:**
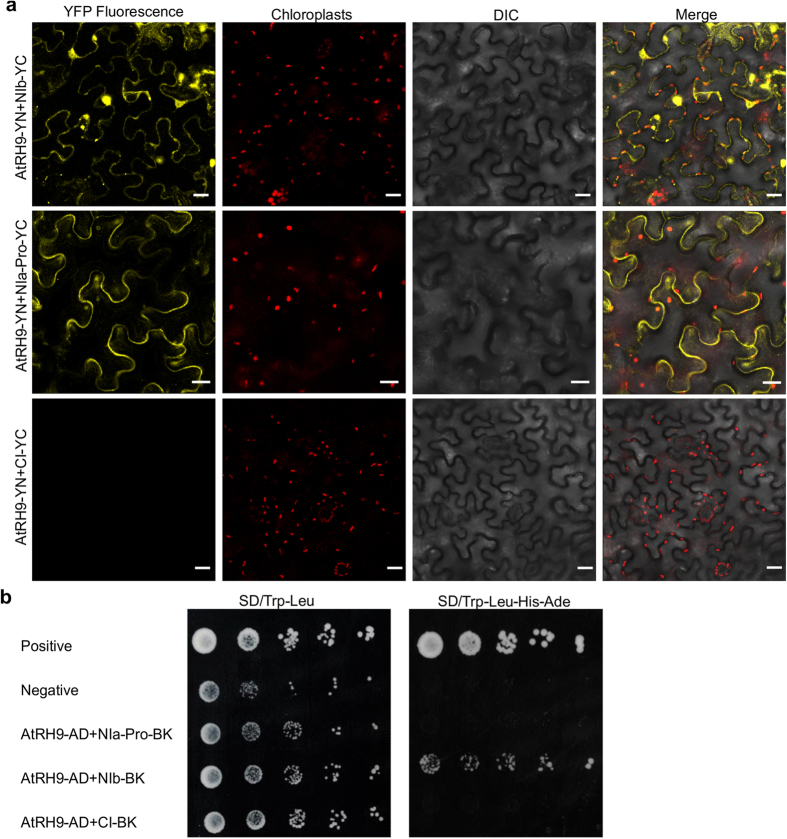
Protein-Protein Interactions between AtRH9 and TuMV Viral Proteins. (**a**) BiFC assay for interactions between AtRH9 and TuMV NIb, NIa-Pro. *N. benthamiana* leaves were co-agroinfiltrated with constructs expressing AtRH9 with TuMV NIb, NIa-Pro and CI fused to the N- and C- terminal half of YFP, respectively. The reconstructed YFP fluorescence was recorded 48 hours post agroinfiltration (hpa) by confocal microscopy. DIC, differential interference contrast. Bars, 20 μm. (**b**) Yeast two-hybrid assay for protein-protein interaction between AtRH9 and NIb. Growth of yeast cells co-transformed with pGAD-AtRH9 and pGBK-NIb, pGBK-NIa-Pro or pGBK-CI were placed on synthetic medium lacking Tryptophan and Leucine (SD/Trp-Leu) to confirm the correct transformation. Different dilutions of yeast transformants were spotted onto a high-stringency selective medium (SD/Trp-Leu-His-Ade) to detect positive interactions, respectively. Co-transformation of pGAD-VPg with pGBK-eIF(iso)4E or pGAD-AtRH9 with pGBK empty vector were used as the positive or negative controls, respectively.

To investigate how AtRH9 is recruited to the VRC, we examined if AtRH9 interacts with TuMV viral proteins *in vivo* using the BiFC assay. The *AtRH9* gene and the coding sequence for each of the 11 TuMV viral proteins were introduced into BiFC vectors containing the DNA fragments encoding the N- or C- terminal moiety of YFP. The resulting plasmids were transiently co-expressed in *N. benthamiana* epidermal cells. The YFP signal would be reconstituted when split fluorescent protein segments were brought together as a result of positive interactions between two tested proteins. Yellow fluorescence was visualized when AtRH9 and NIb or NIa-Pro were co-expressed, suggesting AtRH9 interacts with NIa-Pro and NIb (Fig. 4a). No positive interactions were found between AtRH9 and other viral proteins such as CI (Fig. 4a) or in negative controls (Supplemental Fig. 3).

To confirm the interaction of AtRH9 with TuMV NIb and NIa-Pro, yeast two hybrid (Y2H) assays were performed. Positive interaction was detected between AtRH9 and NIb using a high-stringency selective medium (SD/Trp-Leu-His-Ade) (Fig. 4b). However, no interaction was observed between AtRH9 and NIa-Pro (Fig. 4b). Therefore, it is very likely that *Arabidopsis* AtRH9 is recruited to the VRC via interactions with the viral NIb protein.

## Discussion

In this study, a reverse genetic screening was performed to identify AtRHs that are required for TuMV infection. Of the 41 homozygous T-DNA insertion lines disrupting 26 *AtRH* genes, three genes *AtRH9*, *AtRH26* and *PRH75* were identified as their mutants showed less susceptibility to TuMV infection than WT controls. As shown in Fig. 1, the amount of TuMV CP was significantly reduced and the symptoms were less severe in the *atrh9*, *atrh26* and *prh75* T-DNA mutants in comparison with WT plants. In recent years, several AtRHs including AtRH2^34^, AtRH5^34^, and AtRH20^31^, have been documented to function in infection by tombusviruses, whereas AtRH8 is implicated in potyvirus infection. As all these RHs belong to the family of DDXs that have both RNA-binding and helicase activities, it is reasonable for us to suggest that some members in the DDX family are indeed recruited to assist virus infection in plants. However, so far none of these AtRHs has been found to be involved in infections by two different groups of viruses. As indicated above, AtRH20 is implicated in tombusvirus infection^31^. In this study, TuMV infection was not significantly affected in the *atrh20* mutant (Fig. 1), suggesting AtRH20 is not involved in TuMV infection. Therefore, different viruses may recruit different DDXs to support their infections.

To explore the functional role of the identified RHs in TuMV infection, we focused on AtRH9, since it showed the least accumulation of CP in our screen. qRT-PCR analysis of TuMV accumulation in newly-emerged leaves of *atrh9* mutants or in primarily infected cells revealed that viral RNA was drastically reduced in comparison with that in TuMV-infected WT plants (Fig. 2). To further exclude the possibility that other unknown mutations in the *atrh9* mutant are responsible for reduced TuMV replication, *AtRH9* expression was knockdown in WT *Arabidopsis* seedlings using a VIGS vector and then mechanically inoculated with TuMV. TuMV accumulation was also significantly reduced in the newly-emerged systemic leaves (Supplemental Fig. 2). These results strongly suggest that AtRH9 is a host factor for TuMV replication.

AtRH9 (or PMH1) is hypothesized to be a mitochondrial protein and might be involved in RNA metabolism in mitochondria as an RNA chaperon^40^. However, only small amounts of AtRH9 are present in mitochondrial high molecular weight complexes^40^. This is in contrast to the PHH1 homolog PMH2, which is abundantly present in mitochondrial high molecular weight complexes, acts as a posttranscriptional RNA chaperon, and is required for efficient group II intron splicing. AtRH9 does not influence group II intron splicing efficiency^40^. AtRH9 mRNA level is enhanced in response to biotic stress caused by different pathogens^41^. The subcellular localization of AtRH9 was visualized as punctate particles in the cytoplasm, consistent with the assumed mitochondrial distribution^41^. During TuMV infection, however, the distribution pattern of AtRH9 changed and some AtRH9 was apparent in close proximity with chloroplasts (Fig. 3). This observation raised the possibility that AtRH9 is recruited to TuMV VRCs that are associated with chloroplasts^44^. We thus conducted a co-localization study, and demonstrated that AtRH9 co-localizes with 6K2-induced VRCs. These data strongly suggest that AtRH9 is involved in viral replication.

To understand how AtRH9 is recruited for TuMV infection, the BiFC assay and Y2H assay were conducted to test protein-protein interactions between AtRH9 and TuMV viral proteins. The analysis revealed that AtRH9 interacts with NIb, the viral RNA-dependent RNA polymerase in yeast and plant cells and NIa-Pro, the virus-encoded protease in plant cells (Fig. 4). TuMV NIb catalyzes the synthesis of new viral genomic RNA. The NIb protein accumulates predominantly in the nucleus as nuclear inclusions and is also recruited into the cytoplasmic membrane-bound vesicles that house the VRC during viral infection^21^,^23^,^49^. The interaction of AtRH9 with NIb was confirmed by both BiFC and Y2H assays. Therefore, it is possible that AtRH9 participates in viral replication by associating with viral RdRp. TuMV NIa-Pro is also present in the VRCs and is also responsible for catalyzing the cleavage of P3/6K1, 6K1/CI, CI/6K2, 6K2/NIa, NIa/NIb, NIb/CP and the cleavage site between VPg and NIa-Pro domains in NIa protein^44^,^50^. However, no interaction between AtRH9 and NIa-Pro was detected by the Y2H assay. It is possible that the interaction between AtRH9 and NIa-Pro found in the BiFC assay was either transient or mediated by one more host protein that binds to both AtRH9 and NIa-Pro and thus serves as a bridging interactor. Further study is needed to understand how AtRH9 interacts with NIa-Pro in plants.

In conclusion, by screening and analyzing the susceptibility of *Arabidopsis* DDX T-DNA insertion mutants using a TuMV infection assay, we have provided evidence that DDXs AtRH9, AtRH26 and PRH75 are required for TuMV infection. Further, we have demonstrated that AtRH9 is recruited to the TuMV VRC through interactions with NIb and/or NIa-Pro to promote TuMV replication. However, how AtRH9 affects TuMV replication remains to be elucidated. It is possible that AtRH9 may participate in the separation of RNA duplexes during viral genome replication. Alternatively, it is also possible that AtRH9 facilitates the proteolytic cleavage of TuMV polyprotein via its interaction with NIa-Pro. Future experiments are directed towards a better understanding of the exact biological functions of the identified DDXs in the viral infection cycle.

## Materials and Methods

### Plant materials and growth conditions

*Arabidopsis* (*Arabidopsis thaliana*) ecotype Col-0 and *Nicotiana benthamiana* plants were utilized in this study. Plants were maintained in a growth chamber under constant conditions of 60% relative humidity and a day/night regime of 16 h in the light at 22 °C followed by 8 h at 18 °C in the dark. Seeds for *Arabidopsis* T-DNA insertion lines were obtained from the Arabidopsis Biological Resource Center (ABRC) at Ohio State University, Columbus, Ohio, USA. T-DNA insertion information was obtained from the Salk Institute Genomic Analysis Laboratory website (http://signal.salk.edu/). The *Arabidopsis atrh9* mutants have been described previously^40^. The T-DNA insertion sites of *Arabidopsis atrh9* T-DNA insertion lines in the Col-0 background SALK_035421 and SALK_060677 were confirmed by PCR using the primer pairs (SALK_ 035421-LP: TCATAAATGGAAGTGGCGAAG and SALK_ 035421-RP: TCTTGTTGCAACTGATGTTGC or SALK_ 060677-LP: TTCTCATCCACGGTCAAGATC and SALK_ 060677-RP: TGTACAAGAACCCGTTCTTGG).

### Virus materials, inoculation and TuMV infection assay

The pCambiaTunos/GFP plasmid containing the full-length cDNA of the TuMV genome and pCambiaTunos/6K-mCherry having an additional copy of the 6K2-coding sequence tagged with fluorescent protein mCherry between P1 and HC-Pro were as described previously^51^.

TuMV infection assay was carried out to test the susceptibility of *Arabidopsis* T-DNA insertion lines to TuMV infection. The seedlings of *Arabidopsis* WT plants and selected homozygous mutants were inoculated with TuMV either by mechanical inoculation or using agroinfiltration. Plants were inoculated when at the five to six leaf stage of development. Virus was applied to the two oldest leaves by mechanical inoculation. Approximately 1 g TuMV-infected leaf tissue of *N. benthamiana* was harvested as the source of virus material. The tissue was homogenized using a mortar and pestle in 10 mL inoculation buffer (50 mM potassium phosphate buffer, pH 7.5]. Carborundum powder was lightly dusted on plant leaves, followed by a gentle rubbing of the TuMV-containing inoculum over the leave surface to facilitate virus entry. The negative control plants were rubbed with inoculation buffer also as mock inoculations. The TuMV infection assay was repeated at least three times for each T-DNA insertion line. Systemically infected *Arabidopsis* leaves were harvested for ELISA analysis at 10 dpi.

After mechanical inoculation or agroinfiltration^24^, triple antibody sandwich enzyme-linked immunosorbent assay (TAS-ELISA) was performed to quantify virus accumulation level of WT and *Arabidopsis* T-DNA insertion lines at the days indicated. The newly emerged leaves of *Arabidopsis* mutants and WT plants inoculated with TuMV were harvested for ELISA analysis. Leaf tissue was weighed and ground in ELISA sample extraction buffer. TAS-ELISA was conducted with an ELISA kit (Agdia, Elkhart, IN, USA), following the manufacturer’s instructions. Absorbance values were recorded at 405 nm with an iMark microplate reader (Bio-Rad, Mississauga, Ontario, Canada).

To determine TuMV replication in primarily infected cells, *Arabidopsis atrh9* mutants and WT plants, at the 5–7 leaf growth stage, were agroinfiltrated with a TuMV infectious clone containing an independent cases to express mCherry (TuMV-GFP//mCherry) and a TuMV replication-defective clone (TuMV-∆GDD-GFP//mCherry) (OD600, 0.1), respectively^52^. Agroinfiltrated leaf tissues were harvested at 54 hpa. At this time point, viral intercellular movement did not occur^42^. The harvested tissues were subjected to qRT-PCR analysis.

### Plasmid construction and plant transformation

Gateway technology (Invitrogen, Burlington, Ontario, Canada) was used to generate all the plasmid constructs used in this study, unless otherwise stated. Gene sequences were amplified by polymerase chain reaction (PCR) using Phusion High-Fidelity DNA Polymerase (New England Biolabs, Pickering, ON, Canada) for cloning purposes. GoTaq Flexi DNA Polymerase (Promega, Madison, WI, USA) was employed for genotyping and other analysis.

The full-length P1, HC-Pro, P3, 6K1, CI, 6K2, VPg, NIa-Pro, NIb and CP coding regions of TuMV (GenBank accession NC_002509) were obtained by PCR amplification from the pCambiaTunos/GFP infectious clone^51^,^53^. AtRH9 (At3g22310) coding sequence was generated from cDNA derived from mRNAs isolated from *Arabidopsis* leaves. The resulting DNA fragments were purified and transferred into the entry vector pDONR221 or pENTR (Invitrogen) by recombination using BP Clonase (Invitrogen) following the standard conditions and procedures recommended by the supplier^54^. Insertions in the resulting entry clones were verified by DNA sequencing.

For bimolecular fluorescence complementation (BiFC) assay, the full-length coding sequence of *Arabidopsis* AtRH9 and the coding sequences of TuMV NIb, NIa-Pro and CI cistrons were introduced into the BiFC vectors pEarleygate201-YN or pEarleygate201-YC^55^ to produce AtRH9-YN, NIb-YC, NIa-Pro-YC, and CI-YC respectively.

For transient expression analysis in plant cells, the full-length coding sequence of *Arabidopsis* AtRH9 was transferred by recombination into the binary destination vectors pEarleyGate101^56^ to generate plant expression vector for transient expression of AtRH9-YFP (yellow fluorescence protein) or pEarleyGate102 to produce AtRH9-CFP (cyan fluorescence protein), respectively.

For the targeted yeast two-hybrid assay (Y2H) assay, inserts of the resulting intermediate entry clones were further transferred into modified Gateway-compatible vectors pGADT7-DEST (prey) or pGBKT7-DEST (bait)^56^ by recombination using LR Clonase (Invitrogen) to yield pGAD-AtRH9, pGAD-VPg, and pGBK-NIb, pGBK-NIa-Pro, pGBK-CI, pGBK-eIF(iso)4E, respectively.

For *Tobacco rattle virus* (TRV)-based virus induced gene silencing (VIGS), a 110 bp fragment of AtRH9 was amplified from *Arabidopsis* cDNA using the primer pair that contained an EcoRI and BamHI site specific to the 5′ end and 3′ end of the fragments, respectively (AtRH9-EcoRI-F: 5′- CCGGAATTCTGATGTTGCTGCCCGTGGACT-3′ and AtRH9-BamHI-R: 5′- CGCGGATCCCACGACCAGTTCGCCCCGTT-3′). The amplified fragment was digested with EcoRI and BamHI and cloned into the corresponding sites of pTRV2 vector^57^ to generate the vector pTRV2-AtRH9.

### Yeast two-hybrid assay

Yeast two-hybrid assay (Y2H) was performed following the Clontech yeast protocol handbook using the small-scale lithium acetate yeast transformation method^53^. In brief, yeast cells (strain AH109) were plated onto a selective medium lacking tryptophan and leucine (SD-Trp-Leu) to confirm the right transformation and a high-stringency selective medium lacking tryptophan, leucine, histidine, and adenine (SD-Trp-Leu-His-Ade) to detect positive interactions.

### TRV-based virus-induced gene silencing (VIGS)

To suppress *AtRH9* expression by VIGS, a *Tobacco rattle virus*-based vector was used. pTRV1 and pTRV2-AtRH9 were introduced into *Agrobacterium tumefaciens* (*A. tumefaciens*) and co-agroinfiltrated into *Arabidopsis* seedlings at the four-leaf stage. Plants co-agroinfiltrated with pTRV1 and pTRV2 empty vector or pTRV2-PDS were used as controls. Treated plants were mechanically inoculated with TuMV at 12 dpa.

### RT-PCR and Real-time quantitative RT-PCR (qRT-PCR)

Total RNA was extracted from newly emerged leaves of TuMV-infected *Arabidopsis* mutants and WT plants using the RNeasy Plant Mini Kit (Qiagen) and treated with DNase I following the manufacturer’s instructions. Except otherwise indicated, cDNA synthesized by reverse transcription of RNA samples with Superscript III reverse transcriptase (Invitrogen) and an oligo(dT)_12–18_ primer (Invitrogen) was used to determine the mRNA expression levels of target genes as well as for quantifying TuMV accumulation levels at specific time points indicated. *Arabidopsis Actin II* was used as an internal control.

qRT-PCR was conducted using SsoFast Evagreen supermix (Bio-Rad) and analyzed with the CFX96 Real-Time PCR Detection System (Bio-Rad) following the manufacturer’s instructions. Relative transcript abundances were calculated using Bio-Rad CFX Manager software. The expression of the CP gene of TuMV was determined to reflect the virus accumulation level using primer sets TuMV-CP-F (5′-TGGCTGATTACGAACTGACG-3′) and TuMV-CP-R (5′-CTGCCTAAATGTGGGTTTGG-3′). Gene specific primers (AtRH9-F: 5′-TCGTGCTGGAAAGAAAGGAAGCG-3′ and AtRH9-R: 5′-TTCCACAGCAATGCTAGGCAGCTC-3′) were used quantify *AtRH9* expression. *Arabidopsis Actin II* expression level determined with the primer pair (At-Actin2-F: 5′-CACCACAACAGCAGAGCGGGA-3′ and At-Actin2-R: 5′-TCCCACAAACGAGGGCTGGA-3′) was used as a reference to normalize the data. For detection of viral RNA in WT plants and *atrh9* mutants at primarily infected cells, total RNA was purified at 54 hpa and treated with DNase I (Invitrogen) to remove contaminated DNA. The mCherry transcript level determined with the primers mCherry-F (5′-CACTACGACGCTGAGGTCAA-3′) and mCherry-R (5′-TGGTGTAGTCCTCGTTGTGG-3′) was used as an internal control.

For all qRT-PCR analyses, three biological replicates were included and for each biological replicate, three technical repeats were carried out. All results are shown as means of biological replicates with corresponding standard errors.

### Transient expression in *N. benthamiana*

For transient expression analysis in *N. benthamiana* leaves, constructs were generated in Gateway-compatible binary vectors and transformed into *A. tumefaciens* strain GV3101 via electroporation. Four-week-old *N. benthamiana* plants were used for *Agrobacterium-*mediated transient expression. *Agrobacterium* cultures were grown overnight in LB with appropriate antibiotic selection at 28 °C. The *Agrobacterium* cells were harvested by centrifugation, and then resuspended in infiltration buffer (10 mM MgCl_2_, 10 mM MES, pH 5.7 and 150 μM acetosyringone). After incubation for 2 h at room temperature, the culture was diluted to an optical density of 0.5–1.0 at 600 nm (OD600) and agroinfiltrated into leaf tissue under gentle pressure using a syringe barrel^58^. After agroinfiltration, the plants were maintained under normal conditions for 2–4 days before observation. For Bimolecular Fluorescence Complementation (BiFC) assay, *Agrobacterium* carrying the N-terminal and C-terminal of YFP fusion constructs was co-agroinfiltrated into *N. benthamiana* leaves. The reconstitution of YFP signals were monitored at 2–4 dpa as described^46^.

### Confocal microscopy

Confocal microscopy was performed essentially as described previously^59^,^60^,^61^. Fluorescence was visualized at 2–4 dpa using a Leica TCS SP2 inverted confocal microscope (http://www.leica.com/) with an Argon-Krypton laser. Sections from agroinfiltrated leaves were excised and placed between two microscope cover slides with a drop of water. YFP signals were imaged using a 63× water immersion objective at an excitation wavelength of 514 nm, and emissions were collected between 525 and 575 nm. Images of CFP fluorescence were obtained using the same microscope at an excitation wavelength of 458 nm and emissions were collected between 470 and 500 nm. GFP signal was excited at 488 nm and the emitted light was captured at 505 to 525 nm. mCherry fluorescence was excited at 543 nm and the emitted light was captured at 590–630 nm. Light emitted at 630–680 nm was used to record chlorophyll autofluorescence. Data for the different color channels were collected simultaneously. The samples were scanned at a resolution of 512 × 512 pixels. Images were collected with a charge-coupled device camera and analyzed by Leica confocal software.

## Additional Information

**How to cite this article**: Li, Y. *et al.* Recruitment of *Arabidopsis* RNA Helicase AtRH9 to the Viral Replication Complex by Viral Replicase to Promote Turnip Mosaic Virus Replication. *Sci. Rep.* **6**, 30297; doi: 10.1038/srep30297 (2016).

## Supplementary Material

Supplementary Information

## Figures and Tables

**Table 1:** List of homozygous *Arabidopsis atrh* T-DNA insertion lines for TuMV infection assay.

Gene name	Locus	*Arabidopsis*T-DNA insertion lines
RH1	AT4G15850	SALK_049805; CS839540
DRH1	AT3G01540	SALK_063362; SALK_073018
RH9	AT3G22310	SALK_035421; SALK_060677
RH10	AT5G60990	SALK_001503
RH11	AT3G58510	SALK_122885; SALK_138586
RH12	AT3G61240	SALK_024905; SALK_148563
RH16	AT4G34910	CS852120
RH18	AT5G05450	SALK_083512
RH20	AT1G55150	SALK_124308; SALK_114853
RH21	AT2G33730	SALK_100059; CS839970
RH22	AT1G59990	SALK_032399; SALK_065388
RH24	AT2G47330	SALK_087182; SALK_045730
RH26	AT5G08610	SALK_106823; CS832362
RH28	AT4G16630	SALK_012018; SALK_082807
RH30	AT5G63120	CS848715
RH31	AT5G63630	SALK_090068
RH33	AT2G07750	SALK_017083; SALK_119034
RH37	AT2G42520	SALK_099097
RH40	AT3G06480	SALK_056041
RH41	AT3G02065	SALK_020125; CS843411
RH46	AT5G14610	SALK_068359
RH53	AT3G22330	SALK_056387
RH57	AT3G09720	SALK_019721; SALK_143440
PRH75	AT5G62190	SALK_040389; SALK_068401
STRS1	AT1G31970	SALK_062509
STRS2	AT5G08620	SALK_028850
