# Supplementary material for: Recruitment of Arabidopsis RNA Helicase AtRH9 to the Viral Replication Complex by Viral Replicase to Promote Turnip Mosaic Virus Replication
Source: Sci Rep. 2016 Jul 26;6:30297. doi: 10.1038/srep30297 (PMC4960543; doi:10.1038/srep30297)
Supplement: Supplementary Information [file srep30297-s1.pdf]

Supplementary information

Recruitment of Arabidopsis RNA Helicase AtRH9 to the Viral Replication Complex by Viral Replicase to Promote Turnip Mosaic Virus Replication

Yinzi Li[1,2], Ruyi Xiong[1,2], Mark Bernards[2] & Aiming Wang[1,2] *
[1]London Research and Development Centre, Agriculture and Agri-Food Canada, London, Ontario, N5V 4T3, Canada
[2]Depatment of Biology, Western University, London, Ontario, N6A 5B7, Canada

*For correspondence: Aiming.Wang@AGR.GC.CA

Content

Table S1. Primers Used for PCR-Screening of Homozygous *Arabidopsis atrh* T-DNA Insertion Lines.

Figure S1. Genotyping and RT-PCR Analysis of *Arabidopsis atrh9* T-DNA Insertion Lines.

Figure S2. Knockdown of *AtRH9* Expression Affects TuMV Infection in *Arabidopsis*.

Figure S3. Negative Controls for BiFC Assay.

Figure S4. Sequence Alignment of eIF4A and AtRH9 using the CLUSTAL W Program.

Table S1 Primers Used for PCR-Screening of Homozygous *Arabidopsis atrh* T-DNA Insertion Lines.

Primer Name	Primer sequence (5'-3')
SALK_049805-LP	TTACCCATATTGCCACTGGTC
SALK_049805-RP	TTTATGGAACCCTGTCAGTGC
CS839540-LP	AAACGCTGTTGTGTTGCCTAG
CS839540-RP	AAGTGACTGCACGTTACCAGG
SALK_063362-LP	CAGGTTTGAGGGGTTCCTAAG
SALK_063362-RP	AGGGAATCTCAAAAGCTCGAG
SALK_073018C-LP	ATATCGTGGTTGCAACTCCTG
SALK_073018C-RP	TACCTCTGCCACCATAACCAG
SALK_001503-LP	ATCTGTCCACTTTGGTTGTCG
SALK_001503-RP	GCTTGAATTTTGGAAGGGTTC
SALK_122885-LP	CAAGCGAAGCAAGCAAATTAG
SALK_122885-RP	TTGACTAAAACACCCTTTAGTTTTC
SALK_138586-LP	CAAGCGAAGCAAGCAAATTAG
SALK_138586-RP	AACACCCTTTAGTTTTCTTATAAAAGTG
SALK_024905-LP	CTGCTGAACCACCTCATTAGC
SALK_024905-RP	ACCAAGGGATTAGGTGCAAAG
SALK_148563-LP	CTAGGGAGAGATCTCCACAGG
SALK_148563-RP	TCGAGGAATTGACATTCAAGC
CS852120-LP	ACCTCAACAAGGCATACATCG
CS852120-RP	ACGAGAATCAAGCCCTAGCTC
SALK_083512-LP	TTTCAGGTTCATTCATGAGGC
SALK_083512-RP	TTACTAGCATGTGCAAGCGTG
SALK_124308C-LP	TGAAGGGACGTGATCTTATCG
SALK_124308C-RP	TTTCGATTCACCACAAATTCC
SALK_114853C-LP	TCAATCGGAGAAACTGATTCG
SALK_114853C-RP	GATGCCTCTTGCTGTATCTGC
SALK_100059-LP	CTGAGCTTCATGAGGGTTTTG
SALK_100059-RP	TGTCGAAGATAATCGGTTTCG
CS839970-LP	TCTCGCCCTCTATCTCTCTCC
CS839970-RP	GGATGCGTCGTATCTATGAGC
SALK_032399-LP	CAAGATGCTGTTTGGGTTAGC
SALK_032399-RP	ATTCATCAGATGGCTGATTGC
SALK_065388-LP	AGGGTTGGAGAAGAATTTTGC
SALK_065388-RP	CGGATTTTGCTAGTTCTGCAG
SALK_087182-LP	CCAGAACTCACAGATCTTGCC

SALK_087182-RP	AGCTAAAAACGAGCAAGGAGG
SALK_045730-LP	AATATCTTCATTGGCCATCCC
SALK_045730-RP	GTTACCTCAAGGCCAAGAAGG
SALK_106823-LP	TGCGTATGCCTATAGGACCTG
SALK_106823-RP	TGGTGTCCCTGTCTACGTTTC
CS832362-LP	CAAGAAAACGAGAGAGCAAGC
CS832362-RP	CAGAATTCATGGAAGCGAGAC
SALK_012018-LP	TGTTTGTCAGTTTCAGCATCG
SALK_012018-RP	TATACATGATTGGTCCCACCC
SALK_082807-LP	AAATGTCCTTCTTCTCGTGGG
SALK_082807-RP	TCATCAATTTCAGGGATGAGC
CS848715-LP	CGAGGAAAGTATTGCGATGAG
CS848715-RP	TTGCATTGGATAGGCTTGAAC
SALK_090068-LP	TCACACCCTTTAACCTCGTTG
SALK_090068-RP	ATGGGAGATATTGGGGATTTG
SALK_017083-LP	CTGGCATGGCTCAGATTCTAG
SALK_017083-RP	TGGGTAAACATCGTGTTTTGG
SALK_119034-LP	GAACCAGTCATCTCATGGACG
SALK_119034-RP	CGATAAACCCTCGAAAGAACC
SALK_099097-LP	CGGATATCAATAGCAAGCAGC
SALK_099097-RP	TTGCTTTCCGACTTCTCACAC
SALK_056041-LP	GTTCACAAGAAGATCGCTTGC
SALK_056041-RP	GCCTCATTTTGTCACATCTCC
SALK_020125-LP	ATTCCAAAGATGCAACAGGTG
SALK_020125-RP	GTCTGAGGGATGCTCAGAGTG
CS843411-LP	GGAAAGGGACTGCCATTTAAG
CS843411-RP	TCAAAGGTTGGTCCAATGTTC
SALK_068359-LP	TGAGTAATGGACTTGTTCGCC
SALK_068359-RP	TTGCAAATACTGGAGGGTTTG
SALK_056387-LP	TGATGTGATAGACGAGGGAGG
SALK_056387-RP	TAGGTACATCAAGTCCACGGG
SALK_019721-LP	ACCATTATTGCTGCCTTTTCC
SALK_019721-RP	ATAGCCTGTCTTTGGATTGGG
SALK_143440-LP	ATAGCCTGTCTTTGGATTGGG
SALK_143440-RP	TGTTTCTTCTCTTTGGCGTTC
SALK_040389-LP	CTACAGGTCTGGTCCAGATGG
SALK_040389-RP	TTAAGCTTCTCCCTCAAAGGC
SALK_068401-LP	TTCTAATGTCCTTGCCATTGG

SALK_068401-RP	TTAAGCTTCTCCCTCAAAGGC
SALK_062509C-LP	TGTCCTCCCGATTCTGTGTAC
SALK_062509C-RP	ATATGGGTTTCGAGGAACCTG
SALK_028850-LP	TGGCAATCCAGAATGAGTAGG
SALK_028850-RP	AGCTATCTCCGAAAGAAACGC
LB1	GCCTTTTCAGAAATGGATAAATAGCCTTGCTTCC
LB2	GCTTCCTATTATATCTTCCCAAATTACCAATACA
LB3	TAGCATCTGAATTTCATAACCAATCTCGATACAC
LBb1.3	ATTTTGCCGATTTCGGAAC

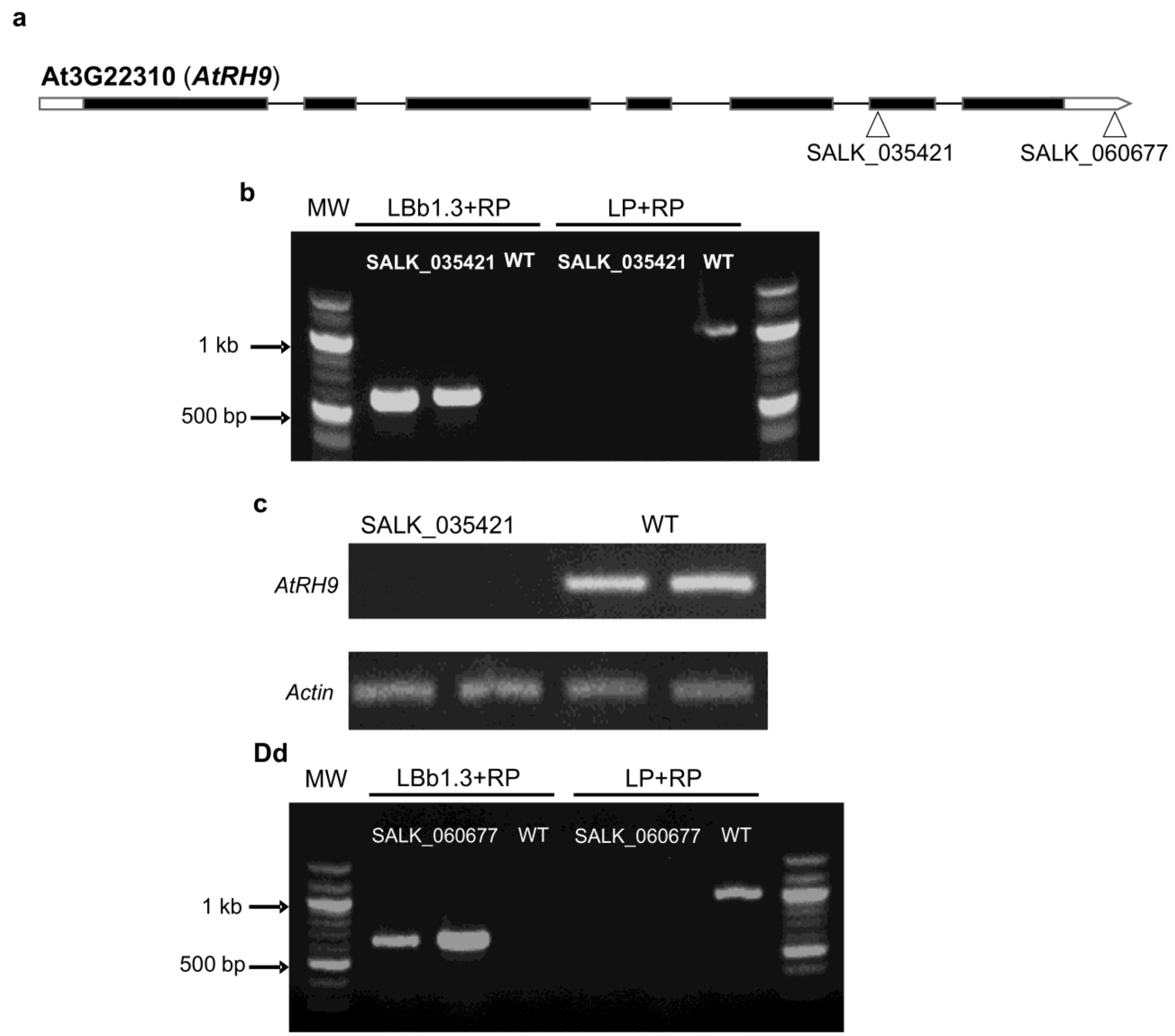

Figure S1. Genotyping and RT-PCR Analysis of *Arabidopsis atrh9* T-DNA Insertion Lines.
a, Schematic characterization of *AtRH9* and T-DNA insertion sites (triangles) in *Arabidopsis* T-DNA insertion mutants. Exons and introns are indicated by boxes and lines respectively. 5' and 3' untranslated regions are shown as open boxes.
b, Screening for homozygous *atrh9* T-DNA insertion lines. PCR was conducted using genomic DNA from *atrh9* (SALK_035421) and WT plants. Two gene-specific primers (LP+RP) were used to detect wild-type genotype. A T-DNA specific primer and a gene-specific primer (LB+RP) were used to amplify a single PCR fragment which represented the pattern of homozygous genotype. WT, wild-type *Arabidopsis*; LP, left genomic primer; RP, right genomic primer; LB, left border primer of the T-DNA insertion.
c, RT-PCR analysis of *AtRH9* expression in *atrh9* mutants and WT plants (SALK_035421). RT-PCR was performed using cDNA derived from leaf tissues of *Arabidopsis atrh9* mutants and WT plants with *AtRH9* specific primers. *Actin2 (Actin)* gene was used as an internal control.
d, Screening for homozygous *atrh9-1* T-DNA insertion line, SALK_060677. A single PCR product was amplified using genomic DNA from the mutant using a T-DNA specific primer and a gene-specific primer (LB+RP).

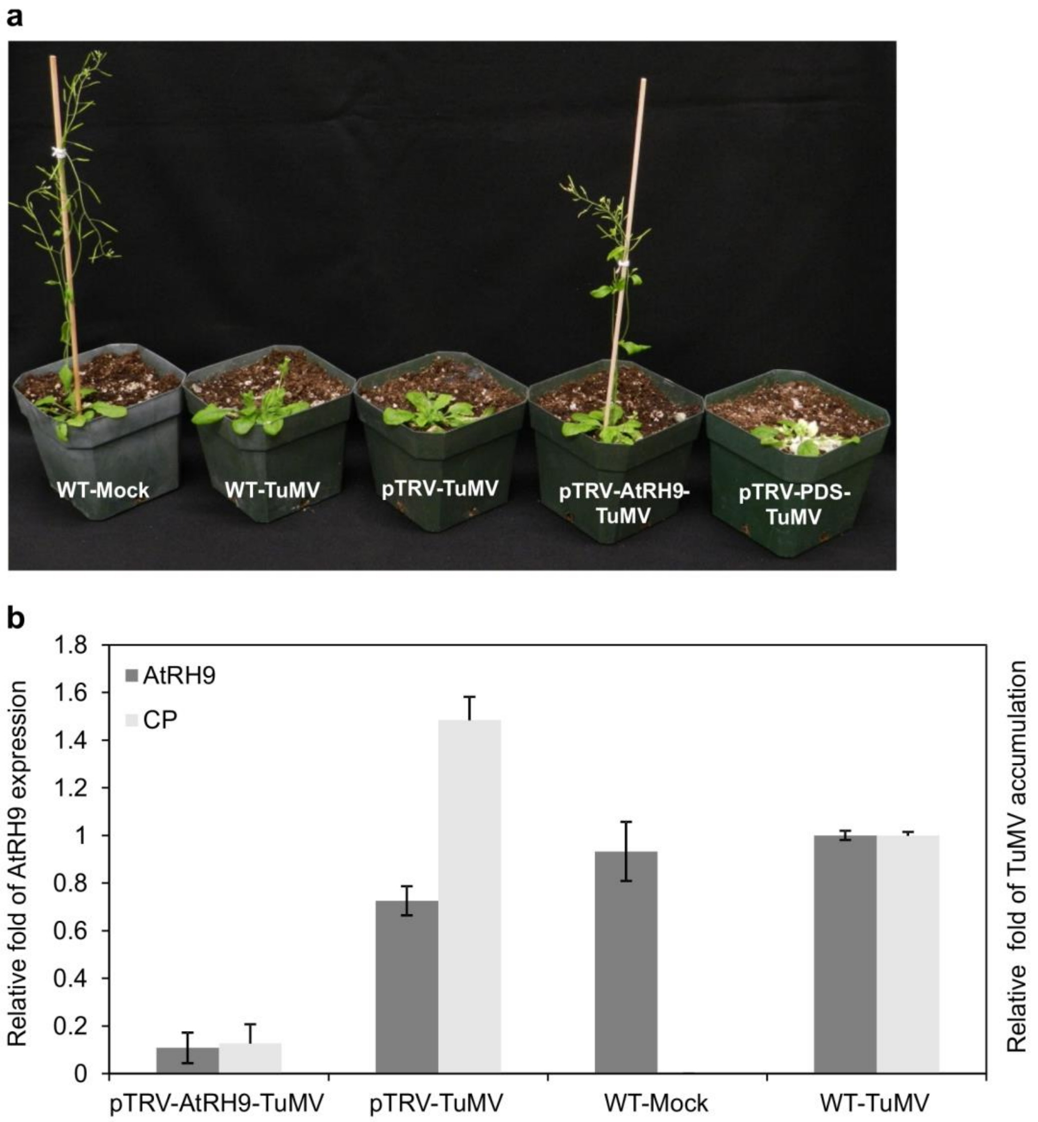

Figure S2. Knockdown of *AtRH9* Expression Affects TuMV Infection in *Arabidopsis*.

a, Phenotypes of TuMV-infected *AtRH9*-knockdown plants, empty VIGS vector-infiltrated plants and *Arabidopsis* WT plants. pTRV-PDS-TuMV, wild-type *Arabidopsis* plants infiltrated with TRV-based VIGS vectors targeting *phytoene desaturase* (*PDS*) to silence and then inoculated with TuMV; pTRV-AtRH9-TuMV, WT infiltrated with TRV-based VIGS vectors targeting *AtRH9* followed by inoculation with TuMV; pTRV-TuMV, WT infiltrated with empty TRV-based VIGS vectors and then inoculated with TuMV; WT-Mock, WT infiltrated with buffer and then inoculated with buffer; WT-TuMV, WT inoculated with TuMV. Images were taken 15 days post inoculation (dpi). TuMV, inoculated with TuMV. Mock, inoculated with buffer.

b, Relative fold changes in TuMV accumulation and expression level of *AtRH9* in *AtRH9*-silenced *Arabidopsis* plants and WT plants. RNA was extracted from leaf tissues for real-time RT-PCR analysis at 15 dpi. Three independent experiments, each consisting of three biological replicates were carried out for quantification analysis. Target genes were normalized against *Actin2* transcripts in each sample. The values are presented as means of fold change relative to the WT plants. Error bars represent standard deviations.

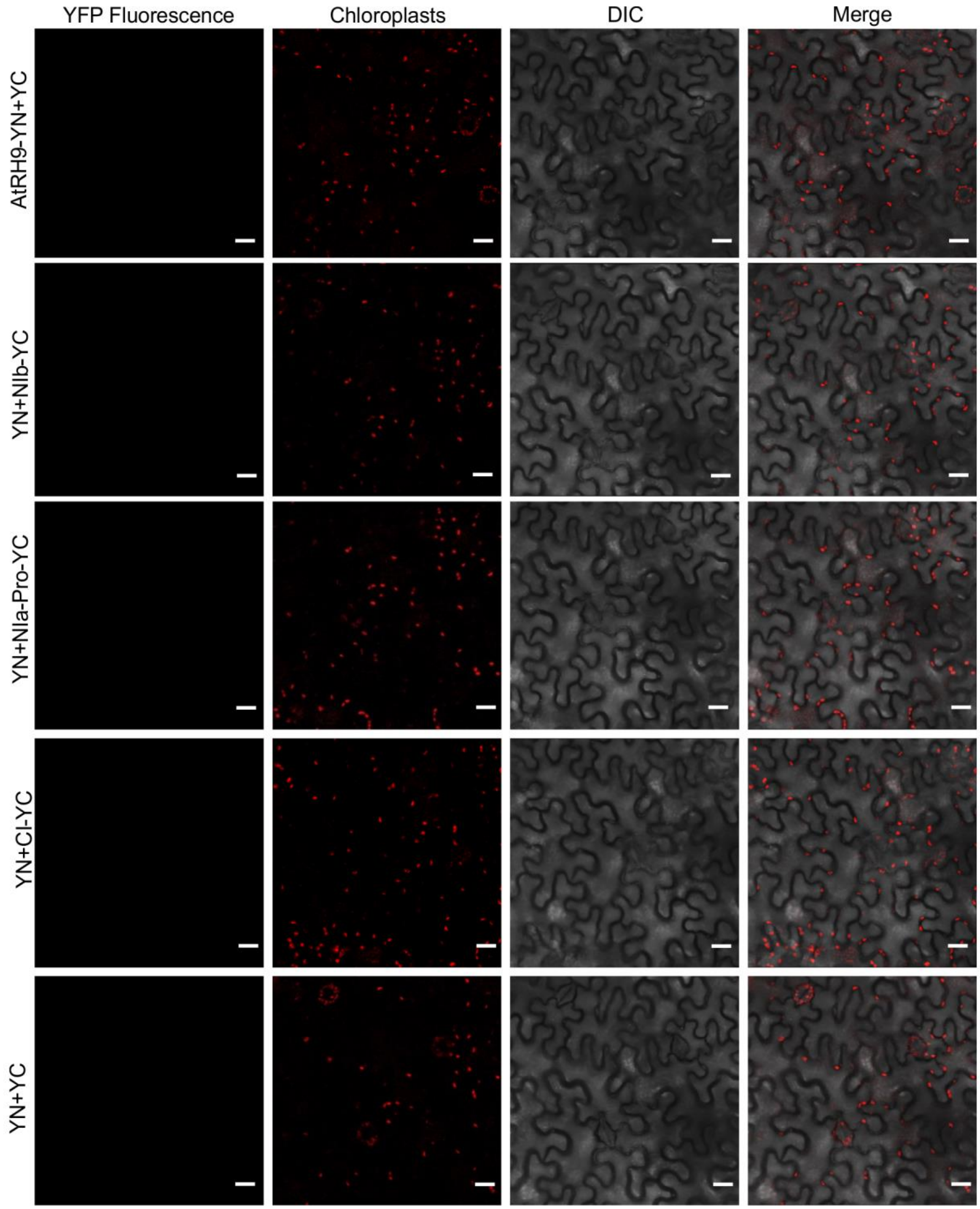

Figure S3. Negative Controls for BiFC Assay.
N. benthamiana leaves were co-agroinfiltrated with constructs expressing AtRH9-YN and YC, YN and NIb-YC, YN and NIa-Pro-YC, YN and CI-YC or YN and YC serving as negative controls. The reconstructed YFP fluorescence was recorded 48 hours post agroinfiltration using a confocal microscopy. No YFP fluorescence signal was observed in all the negative controls displayed. DIC, differential interference contrast. Bars, 20 µm.

```
                                                          Motif Q
eIF4A     --------------------------------VHESFDAMGLQENLLRGIYAYGFEKPSA
AtRH9     ----------------DGGIGDSESVGSSGGGDGLAIADLGISPEIVKALKGRGIEKLFP

                            Motif I
eIF4A     IQQRGIVPFCKGLDVIQQAQSGTGKTATFCSGVLQQLD-FS-----LIQC--QALVLAPT
AtRH9     IQKAVLEPAMEGRDMIGRARTGTGKTLAFGIPIIDKIIKFNAKHGRGKNP--QCLVLAPT

         Motif Ia                                           Motif Ib
eIF4A     RELAQQIEKVMRALG---DYLGVKVHACVGGTSVREDQRILQ-AGVHVVVGTPGRVFDML
AtRH9     RELARQVEKEFRESA---PSLD--TICLYGGTPIGQQMRELN-YGIDVAVGTPGRIIDLM

                        Motif II                          Motif III
eIF4A     KRQ---SLRADNIKMFVLDEADEMLSRGFKDQIYDIFQLLPPKIQVGVFSATMPPEALEI
AtRH9     KRG---ALNLSEVQFVVLDEADQMLQVGFAEDVEIILQKLPAKRQSMMFSATMPSWIRSL

eIF4A     TRKFMSKPVRI-LVKRD--ELTLEGI-KQFYVNVEKEEWKLETL-CDLY-ETLA---ITQ
AtRH9     TKKYLNNPLTI-DLVGDSDQKLADGI-TMYSIAAD--SYGRASIIGPLVKEHGK---GGK

            Motif IV
eIF4A     SVIFVNTRRKVDWLTDKMRSRDHTVSATHGDMDQNTRDIIMREFRSGSSRVLITTDLLAR
AtRH9     CIVFTQTKRDADRLAFGL-AKSYKCEALHGDISQAQRERTLAGFRDGNFSILVATDVAAR

         Motif V            Motif VI
eIF4A     GIDVQQVSLVINFDLPTQPENYLHRIGRSGRFGRKGVAINFVTRDDERMLC-LRTWPICC
AtRH9     GLDVPNVDLVIHYELPNNTETFVHRTGRTGRAGKKGSAILIHGQDQTRAVK-MIEKEVGS
```

Figure S4. Sequence Alignment of eIF4A and AtRH9 using the CLUSTAL W Program. All the conserved motifs (Q, I, Ia, Ib, II, III, IV, V, VI) of DEAD-box RNA helicase were shown in boxes. The accession numbers of the aligned protein sequences are eIF4A (At3g13920) and AtRH9 (At3g22310).
